# Cost-Effective Mechanical Aggregation of Cardiac Progenitors and Encapsulation in Matrigel Support Self-Organization in a Dynamic Culture Environment

**DOI:** 10.3390/ijms232415785

**Published:** 2022-12-13

**Authors:** Tiago P. Dias, Sandra N. Pinto, Sandra Carvalho, Tiago G. Fernandes, Fábio Fernandes, Maria Margarida Diogo, Maria C. Peleteiro, Manuel Prieto, Joaquim M. S. Cabral

**Affiliations:** 1 iBB—Institute for Bioengineering and Biosciences and Department of Bioengineering, Instituto Superior Técnico, Universidade de Lisboa, Av. Rovisco Pais, 1049-001 Lisbon, Portugal; 2 Associate Laboratory i4HB—Institute for Health and Bioeconomy at Instituto Superior Técnico, Universidade de Lisboa, Av. Rovisco Pais, 1049-001 Lisbon, Portugal; 3 CIISA, Centre for Interdisciplinary Research in Animal Health, Associate Laboratory for Animal and Veterinary Sciences (AL4AnimalS), Faculdade de Medicina Veterinária, Universidade de Lisboa, Av. da Universidade Técnica, 1300-477 Lisbon, Portugal

**Keywords:** manual aggregation, cardiac differentiation, WNT signaling modulation, dynamic culture, Matrigel encapsulation, self-organization

## Abstract

Human iPSC-derived self-organized cardiac tissues can be valuable for the development of platforms for disease modeling and drug screening, enhancing test accuracy and reducing pharmaceutical industry financial burden. However, current differentiation systems still rely on static culture conditions and specialized commercial microwells for aggregation, which hinders the full potential of hiPSC-derived cardiac tissues. Herein, we integrate cost-effective and reproducible manual aggregation of hiPSC-derived cardiac progenitors with Matrigel encapsulation and a dynamic culture to support hiPSC cardiac differentiation and self-organization. Manual aggregation at day 7 of cardiac differentiation resulted in 97% of beating aggregates with 78% of cTnT-positive cells. Matrigel encapsulation conjugated with a dynamic culture promoted cell migration and the creation of organized structures, with observed cell polarization and the creation of lumens. In addition, encapsulation increased buoyancy and decreased coalescence of the hiPSC-derived cardiac aggregates. Moreover, VEGF supplementation increased over two-fold the percentage of CD31-positive cells resulting in the emergence of microvessel-like structures. Thus, this study shows that the explored culture parameters support the self-organization of hiPSC-derived cardiac microtissues containing multiple cardiac cell types. Additional stimuli (e.g., BMP) in long-term scalable and fully automatized cultures can further potentiate highly structured and mature hiPSC-derived cardiac models, contributing to the development of reliable platforms for high-throughput drug screening and disease modeling.

## 1. Introduction

Human induced pluripotent stem cell (hiPSC)-derived cardiac tissues have the potential to be an impactful tool for drug screening and disease modeling, unveiling disease mechanisms and speeding up the development of new treatments, while increasing safety and reducing pharmaceutical industry financial burden caused by drug withdrawals from the market due to hidden side effects associated with cardiotoxicity [1,2,3]. Consequently, a high demand is present for the development of systems that support the differentiation, self-organization, and maturation of hiPSC-derived cardiac tissues towards more complex tissues that can fully mimic the heterogeneity and functionality of the human mature cardiac tissue.

Exploring hiPSCs’ self-organization capabilities can allow for the development of organoids with the potential to closely resemble adult tissues [4,5,6,7]. The presence of a multitude of cell types, belonging to the tissue of interest, and the interactions between the different cell types and self-organized structures provide additional functionality that increases the reliability to use these derived microtissues for both drug screening and disease modeling [8]. Formation of organoids is often initiated with an aggregation step, which creates a niche environment that promotes the action of morphogenetic processes conditioned by the formation of gradients of nutrients, metabolites, supplemented factors, and autocrine growth factors [9,10,11]. These gradients stimulate self-organization by providing a vector that induces differentiation heterogeneity, cell migration, polarization, and the development of complex structures [9,10,11]. Thus, aggregate size is critical for the differentiation outcome, and in particular for cardiac differentiation [12,13,14,15]. Frequently, for better control, aggregation is performed using specialized commercially available microwells [13,15,16,17]. Nevertheless, the use of these culture plates increases costs since their reutilization is ill-advised and can impact overall yield and system scalability.

In addition, organogenesis is often performed in static conditions [18,19,20,21,22,23]. However, the culture of hiPSC aggregates in static conditions presents several challenges, namely low access to nutrients and poor metabolic waste removal in the inner most parts of the organoids. These limiting conditions can inhibit organoid development and maturation during long-term cultures by constraining organoid growth in size and, ultimately, leads to the loss of viability and functionality [24,25]. Therefore, seamless integration of a dynamic culture with organogenesis is essential to take full advantage of the self-organization capabilities of hiPSCs and leads to the development of more reliable models that can closely resemble fully developed tissues.

Herein, we demonstrate a cost-effective aggregation and differentiation system integrated with a dynamic culture for the development of hiPSC-derived cardiac aggregates showing self-organization capabilities and containing multiple cardiac cell types. Cost-effective, reliable, and reproducible aggregation was performed at day 7 by mechanically detaching the hiPSC-derived monolayer after initiating cardiac differentiation by WNT signaling modulation. Compared with non-encapsulated aggregates, encapsulation in Matrigel conjugated with further culture in dynamic conditions improved self-organization with observed cell migration and the development of complex structures. Additional supplementation with VEGF lead to an increase in CD31-positive cells and the development of microvessel-like structures.

## 2. Results

### 2.1. Establishment of Culture Conditions for the Generation of 3D Cardiac Microtissues from hiPSCs

**Figure 1:**
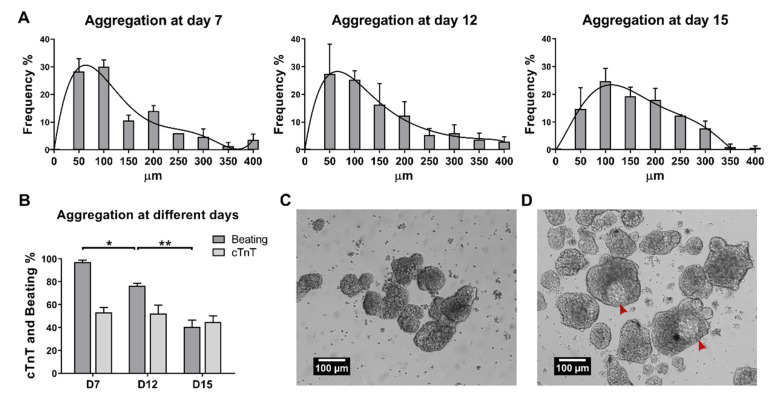
Manual induction of aggregation is reliable and cost-effective when performed at day 7. (**A**) Aggregate size distribution for mechanical aggregation performed at day 7, day 12, or day 15 of cardiac differentiation. Aggregation at day 7 and day 12 promoted a higher frequency of aggregates with a size of 50 and 100 µm. At day 15, aggregation yielded a higher variance population. The lines correspond to centered sixth order polynomials that model size distribution. Error bars, SEM, *n* = 3. (**B**) Percentage of beating aggregates and percentage of cells expressing cTnT (determined by flow cytometry) at day 18 of cardiac differentiation. Aggregation at day 7 resulted in an average of 97% aggregates with a beating phenotype and 53% cTnT-positive cells. Error bars, SEM, *n* = 3. * *p*-value < 0.05, ** *p*-value < 0.01, (ANOVA with Tukey’s test). (**C**,**D**) Aggregates at day 10 (**C**) and day 15 (**D**) of differentiation after aggregate induction at day 7. Some aggregates appeared to develop structures with different depths (red arrows). Scale bar: 100 µm.

We began with the goal of overcoming the use of specialized, commercially available microwells by optimizing manual aggregation. We induced hiPSC cardiac differentiation in monolayer using the WNT signaling modulation protocol [26]. Then, we tested different time points to induce aggregation of the hiPSC-derived monolayer. Aggregation was induced manually at day 7, day 12, and day 15. Manual aggregation at day 7 showed the most reproducible result compared with aggregation at days 12 and 15, with the majority of the aggregate diameters ranging between 50 and 100 µm (Figure 1A). Homogeneous populations of size-controlled aggregates are important to reduce variability, in part by decreasing necrosis in the aggregates’ center, due to low nutrient diffusion, which impacts cardiac differentiation and protocol reproducibility [12,13,14,27,28]. At day 15, aggregate formation was difficult to perform due to the increased structural integrity of the cardiac microtissue, resulting in a more heterogeneous population with an increasing number of larger aggregates (Figure 1A).

In addition, manual aggregation at day 7 resulted in a significantly higher number of aggregates with a beating phenotype; 97% compared to 76% and 41% for day 12 and day 15, respectively (Figure 1B). Nevertheless, there were no significant differences in the percentage of cTnT-positive cells at day 18 for the different time points of manual aggregation. Furthermore, aggregation at day 7 resulted in some aggregates showing the formation of structures with different depths at day 15 of differentiation (Figure 1C,D, red arrows, and Video S1).

Overall, the results showed that manual aggregate induction at an earlier stage of differentiation, day 7, yielded more homogenous populations of aggregates with suitable sizes and increasing numbers of beating aggregates.

**Figure 2:**
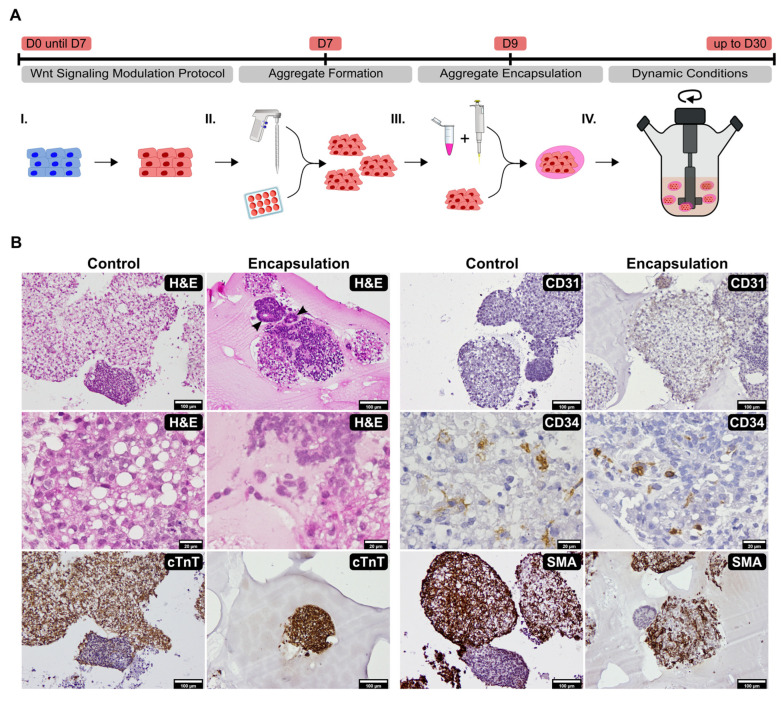
Matrigel encapsulation and a dynamic culture environment promotes cell migration and organization. (**A**) Scheme describing the experimental procedure. The WNT signaling modulation protocol was initiated in monolayer (I) with aggregate formation performed manually at day 7 (II). Encapsulation of aggregates with Matrigel was performed at day 9 (III) with encapsulated aggregates being cultured in dynamic conditions up to 30 days (IV). (**B**) H&E staining and immunohistochemistry panel of anti-cTnT, anti-CD31, anti-CD34, and anti-SMA markers of control and encapsulated aggregates after culture in dynamic conditions (*n* = 3). H&E staining highlighted organized structures in encapsulated aggregates, in some cases showing cell polarization with the creation of lumens (black arrows) and cell migration into the Matrigel matrix. Scale bar: 100 µm, top and bottom, 20 µm mid.

Next, we hypothesized that encapsulating the aggregates in Matrigel would provide a niche that could potentially promote increased self-organization, a method already shown to increase neural [5], gut [7,29,30,31], liver [32], retinal [33], lung [34], and pancreatic [35] organogenesis. In addition, we cultured aggregates with and without Matrigel encapsulation in dynamic conditions, using 15 mL spinner flasks, to further increase the diffusion of nutrients, dissolved oxygen, and metabolites, allowing for minimization of gradients and an increase in pH homogeneity, thus providing more suitable culture conditions for the aggregates [14,36]. Encapsulation was manually performed at day 9 and aggregates were cultured under dynamic conditions for up to 30 days (Figure 2A and Video S2). Non-encapsulated aggregates (control) commonly showed aggregate coalescence whereas encapsulated aggregates showed increased buoyancy and did not coalesce (Figure 2B).

In addition, encapsulated aggregates showed enhanced self-organization with H&E staining, highlighting organized structures with cell polarization and the creation of lumens (black arrows) and cell migration into the Matrigel matrix (Figure 2B, Encapsulation H&E). Contrarily, non-encapsulated aggregates showed increased vacuolization—a sign of lipid accumulation, likely due to hypoxic conditions created by the coalescing aggregates—and cellular apoptosis (Figure 2B, Control H&E). cTnT immunohistochemistry highlighted that almost all aggregates contained a high number of positive cardiomyocytes (Figure 2B, Encapsulation cTnT) although with a few exceptions, especially in the control (Figure 2B, Control cTnT). CD31 labelling was sparse, mostly present in aggregates’ periphery (Figure 2B, Encapsulation CD31) and mainly detected in encapsulated samples (Figure 2B, Control CD31), while CD34 positivity was observed inside of both control and encapsulated aggregates (Figure 2B, Control and Encapsulation CD34). Smooth muscle actin (SMA) immunohistochemistry showed positive labelling throughout the majority of aggregates, although with a few aggregates showing weaker or absent immunomarking (Figure 2B, Control and Encapsulation SMA).

Overall, these results showed aggregate cellular composition diversity with the presence of cardiomyocytes and non-cardiomyocyte cardiac cells such as endothelial, endothelial progenitors, and smooth muscle/myofibroblast cells. In addition, Matrigel encapsulation provided an enriched environment for aggregate culture and when conjugated with dynamic conditions it seems to increase cardiac microtissues’ complexity and self-organization capabilities.

### 2.2. Impact of VEGF Supplementation and Cell Aggregation on the Development of Microvessel-like Structures and Cardiomyocyte Functionality of Cardiac Microtissues

To increase the content of endothelial progenitors in cardiac microtissues and vascularization, VEGF supplementation, which is associated with the promotion of endothelial progenitor generation during differentiation and angiogenesis, was added (50 ng/mL) at day 7, during manual aggregation, and at day 10 of aggregate maturation.

**Figure 3:**
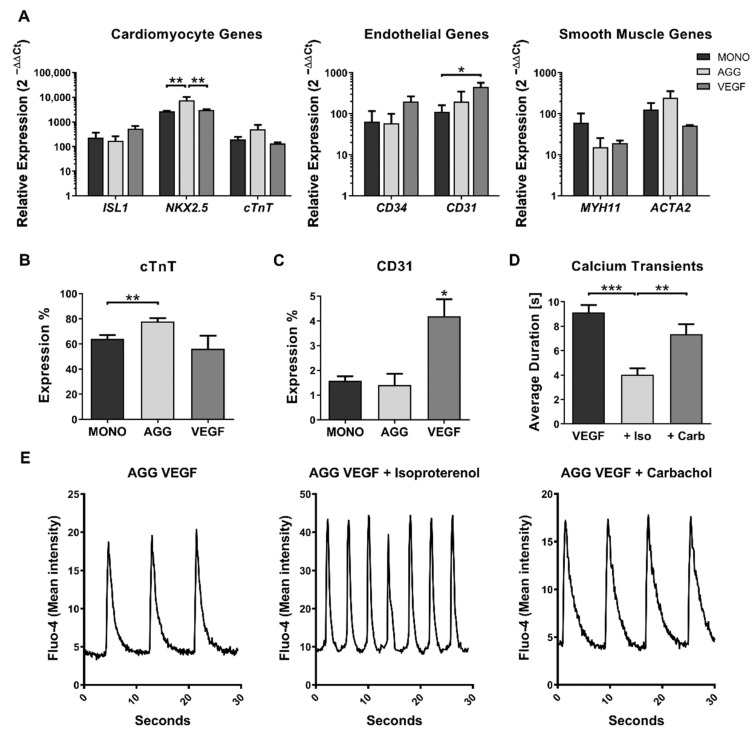
Aggregate characterization by analysis of lineage markers using real-time PCR, flow cytometry, and calcium transient microscopy. (**A**) Analysis of lineage markers gene expression by real-time PCR. Most genes expression was not significantly different between the monolayer (MONO), aggregates (AGG), and aggregates supplemented with VEGF (VEGF). Addition of VEGF significantly increased the expression of *CD31*. Error bars, SEM, *n* = 3. * *p*-value < 0.05, ** *p*-value < 0.01 (2-way ANOVA with Tukey’s test). (**B**) Flow cytometry showed an increase in cTnT-positive cells for aggregates without VEGF compared with the monolayer and aggregates supplemented with VEGF. Error bars, SEM, *n* = 6 for MONO, *n* = 4 for AGG and *n* = 3 for VEGF. ** *p*-value < 0.01 (Welch’s *t*-test). (**C**) CD31 protein expression increased over two-fold when a double dosage of VEGF was supplemented compared with the monolayer and aggregates without VEGF. Error bars, SEM, *n* = 7 for MONO, *n* = 6 for AGG, and *n* = 3 for VEGF. * *p*-value < 0.05 (Welch’s *t*-test). (**D**) Calcium transient analysis of aggregates supplemented with VEGF. The average time between calcium transients demonstrates a strong statistically significant decrease from 9.1 s to 4.0 s when isoproterenol (+Iso) was used. Carbachol (+Carb) showed no statistically significant effects (7.3 s). Error bars, SEM, *n* = 10 for VEGF, *n* = 8 for +Iso, *n* = 7 for +Carb. ** *p*-value < 0.01, *** *p*-value < 0.001 (Welch’s *t*-test). (**E**) Representative calcium transient profiles. Left, aggregates supplemented with VEGF (8.5 s, SD ± 0.1), middle, when 1 µM isoproterenol was supplemented (4.0 s, SD ± 0.1), and right, when 1 µM carbachol was supplemented (8.0 s, SD ± 0.1).

To characterize the impact of VEGF and aggregation, we analyzed the gene expression of markers for cardiomyocytes (*ISL1*, *NKX2.5* and *cTnT*), endothelial (*CD34* and *CD31*), and smooth muscle cells (*MYH11* and *ACTA2*) for the protocol of monolayer differentiation (MONO), cell aggregation at day 7 (AGG), and cell aggregation at day 7 plus supplementation with VEGF (VEGF) (Figure 3A). Most genes were similarly expressed among the three conditions. *NKX2.5* expression was statistically significantly increased in aggregates compared with MONO and VEGF supplementation (Figure 3A). A slight increase in *cTnT* gene expression could also be observed for aggregates without VEGF supplementation. In addition, VEGF supplementation promoted a statistically significant increase in the expression of *CD31* (Figure 3A). A slight increase in *CD34* could also be observed when VEGF supplementation was used.

Flow cytometry analysis of the cTnT marker (Figure 3B) showed an increase in the percentage of cells expressing this marker in aggregates, with 78% cTnT-positive cells, when compared to monolayer, with 64%, which is in accordance with the gene expression observed for the cardiomyocyte panel (Figure 3A). In addition, in agreement with the PCR results, VEGF supplementation resulted in a significantly increased expression of CD31, over two-fold, compared with both the monolayer and aggregation without VEGF supplementation (Figure 3C).

To test the functionality of aggregates supplemented with VEGF, calcium transients were measured with or without supplementation of isoproterenol, a β-adrenergic receptor agonist that increases heartbeat frequency, or carbachol, a muscarinic agonist that slows contraction frequency. Supplementation of isoproterenol (+Iso) highly increased calcium transient average frequencies, reducing on average the time between transients from 9.1 to 4.0 s (Figure 3D). However, some aggregates lost the contractile phenotype, which indicates some degree of immaturity, which is expected at this stage of differentiation [37]. When carbachol (+Carb) was supplemented, large masses of coalesced aggregates seemed to synchronize their beating phenotype, although no significant effect was detected regarding the calcium transient duration which was on average 7.3 s (Figure 3D). Figure 3E shows representative calcium transient profiles for aggregates supplemented with VEGF and those exposed to isoproterenol or carbachol.

**Figure 4:**
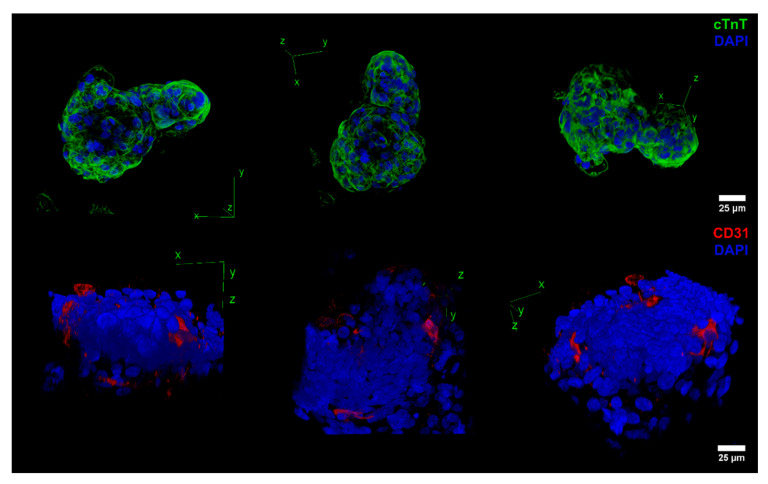
Confocal fluorescence microscopy of aggregates supplemented with VEGF showed the presence of microvessel-like structures. Representative 3D projection of aggregates supplemented with VEGF stained with cTnT and DAPI or CD31 and DAPI. Supplementation of VEGF maintained tissue complexity and cTnT expression, while resulting in the development of CD31-positive microvessel-like structures. Scale bar: 25 µm.

In addition, 3D projections were performed for the aggregates using the z-stack capabilities of confocal microscopy (Figure 4). Supplementation of VEGF maintained tissue structural complexity and cTnT expression (Figure 4 cTnT panel). In addition, VEGF stimulated the appearance of CD31-positive microvessel-like structures in accordance with the flow cytometry results (Figure 4 CD31 panel). These structures were observed in most aggregates exposed at day 7 and day 10 to 50 ng/mL of VEGF.

Overall, these results showed some degree of functional response of the hiPSC-derived cardiac microtissues, although larger maturation times in dynamic conditions may be needed to increase the degree of functional maturation. In addition, supplementation of VEGF successfully promoted the development of microvessel-like structures.

## 3. Discussion

Cost-effective and scalable systems capable of maximizing the organogenesis potential of hiPSCs are important for the broad implementation of hiPSC-derived organoid models as a novel tool in pre-clinical drug development. However, many of the systems currently used to develop organoids still rely in costly cell aggregation methods and static conditions, which limits hiPSC-derived organoids from reaching their full potential namely in terms of industrial applications. The goal of this work was the development of an integrated process for the production of cardiac microtissues from hiPSCs including a reliable method of aggregation followed by a dynamic culture system to support hiPSC cardiac differentiation and self-organization. First, we implemented a method of manual aggregation after the initial steps of cardiac commitment of hiPSCs in monolayer conditions to bypass the use of costly commercial aggregation platforms with low scalability potential. Secondly, we encapsulated the hiPSC-derived cardiac aggregates in Matrigel and cultured the aggregates in dynamic conditions. In addition, we incorporated VEGF supplementation to support and increase cellular composition diversity, namely in terms of endothelial progenitors. Our results showed that coupling reproducible manual aggregation with Matrigel encapsulation, dynamic culture, and VEGF supplementation generated cardiac microtissues showing the self-organization of multiple cell types and the development of complex structures.

Manual aggregation originated, with high reproducibility, almost 100% of beating aggregates and a population with a size range compatible with cardiac differentiation, which is on par with previously published platforms [15,38,39]. Moreover, compared with previously published platforms, the resulting microtissues presented an increased cellular heterogeneity, with a lower percentage of cTnT-positive cells conjointly with the presence of other cardiac cell types. Thus, when compared with other differentiation platforms that maximize cTnT-positive cells and originate microtissues with more homogenous cell populations, our platform might be advantageous if the final purpose is organogenesis. In addition, our platform generates aggregate populations with lower sizes, which reduces the negative contribution of oxygen gradients since oxygen diffusion is predictably limited to less than 150 μm [40]. In addition, manual aggregation allowed us to bypass the use of specialized, commercially available microwells, which permitted us to reduce costs and labor intensity. Furthermore, the procedure of mechanical aggregation can be fully automatized and integrated with other essential steps of hiPSC culture using a robotic platform, either by optimizing different EDTA incubation times [41] or by using a scraper attachment [42], thus facilitating the manufacture of standardized, cost-effective, and efficient high-throughput platforms for broader applications. Moreover, integration with chemically defined culture systems can further increase reproducibility and it is important to explore potential translational applications. In addition, methodologies that can overcome the expansion limitations of cardiac progenitor cells or immature hiPSC-derived cardiomyocytes [43], such as the use of FOXO inhibitors [44], could contribute to further improving the overall scalability of the system and lowering costs.

During the period in which this work was in preparation, several studies were published highlighting the generation of complex, highly structured hiPSC-derived cardiac organoid models, including multilineage organoids [19,20,21,22,23]. In this study, we observed the presence of multiple cell types and distinct structural organization with the addition of VEGF further increasing the display of CD31-positive structures. However, our work has several limitations in comparison with these publications since, besides VEGF supplementation, we did not incorporate additional factors to the WNT signaling biphasic modulation protocol or pursue longer maturation periods. Nonetheless, our main focus was to evaluate the introduction of a dynamic culture and Matrigel encapsulation. None of the works mentioned explored dynamic conditions for the long-term culture of the cardiac organoids developed. Our work suggests that these highly structured cardiac organoid models might further benefit from integration with a dynamic culture in particular when organoids reach a size that significantly decreases nutrient diffusion and the creation of detrimental gradients. Furthermore, our work showed that Matrigel encapsulation had a significative impact on cell migration and self-organization, provided increased buoyancy, and avoided aggregate coalescence, which facilitated culture in dynamic conditions. Among the previously mentioned studies, only two studies incorporated encapsulation with Matrigel [19,21]. Mills et al. used a mixture of hPSC-derived cardiomyocytes with collagen and Matrigel to create gel surrounding PDMS exercise poles [19]. Drakhlis et al., similarly to our protocol, relied only in the WNT signaling modulation for inducing cardiac differentiation and showed that Matrigel encapsulation was indispensable to promoting the organogenesis process that leads to early cardiomyogenesis patterning and the proper development of their cardiac organoid model [21]. Hofbauer et al. showed that substitution of Matrigel with xeno-free and chemically-defined laminins 521/511 can support the formation of a cardiac organoid model that recapitulates chamber-like morphogenesis; although, the authors found that exogenous ECM was not required when differentiation was performed entirely in a non-adherent culture [23]. In addition, the multilineage cardiac–gut model presented by Silva et al. did not require the use of any exogenous ECM [20]. Thus, the necessity of exogenous ECM and the creation of a microenvironment might be protocol-specific and its impact on reproducibility and maturation still needs to be fully assessed.

In summary, we integrated a dynamic culture and Matrigel encapsulation with a reliable and cost-effective aggregation method to generate cardiac microtissues. Our study showed that conjugation of Matrigel encapsulation with a dynamic culture stimulates cell migration and the creation of lumens with the presence of multiple cardiac cell types. Additional stimuli, such as supplementation with BMP [45], and long-term culture in a dynamic environment might contribute to further develop highly structured and mature hiPSC-derived cardiac organoid models, in line with recently published works. Incorporation of hiPSC organogenesis into scalable and fully automatized systems will further support the development of cost-effective organoid platforms with a degree of maturation and complexity required for reliable high-throughput drug screening and disease modeling.

## 4. Materials and Methods

### 4.1. Human Induced Pluripotent Stem Cell Culture

In this work, the hiPSC cell line iPS-DF6-9-9T.B, purchased from WiCell Bank, was used. This cell line is vector free and was derived from foreskin fibroblasts with a karyotype 46, XY. The hiPSC culture was performed using mTeSR1 medium (STEMCELL Technologies, Vancouver, BC, Canada) in 6-well tissue culture plates coated with Matrigel (BD Biosciences, Franklin Lakes, NJ, USA) diluted 1:30 in DMEM/F12. The medium was changed daily. Enzyme-free passaging was performed using an EDTA (Thermo Fisher Scientific, Waltham, MA, USA) solution diluted in PBS at a concentration of 0.5 mM. The cells were incubated for 5 min with EDTA at room temperature and flushed with culture medium. Splits from 1:3 to 1:8 were usually performed. For cell counting, a sample of 100 µL was incubated in 400 µL of Accutase (Thermo Fisher Scientific) for 7 min at room temperature and the samples were diluted in trypan blue. Phase contrast images were obtained using a Leica DMI 3000B microscope and a Nikon DXM 1200 digital camera.

### 4.2. Human iPSC Cardiomyocyte Differentiation, Aggregation, and Encapsulation

Human iPSCs were seeded at a density of 1 × 10^5^ cells/cm^2^ in 12-well tissue culture plates and maintained in pluripotent conditions with daily medium changes. At around 95% confluency, hiPSC cardiac differentiation was induced by adapting the WNT signaling modulation protocol previously described [46].

Aggregation at days 7, 12, or 15, was promoted by exposing differentiating hiPSCs to 0.5 mM EDTA for 3 min. The cells were then mechanically detached using a 5 mL pipette and aggregate-size thinned using a P1000 micropipette. The medium was changed every three days afterwards using RPMI/B27 with insulin medium. In experiments using VEGF, the aggregates were supplemented with 50 ng/mL of VEGF A (R&D) at day 7 and day 10. Aggregate sizes were calculated using an ocular micrometer. Beating aggregates were manually counted. 

Encapsulation was promoted by selecting an aggregate with a P20 micropipette and involving it in Matrigel with an approximate dilution of 1 to 1 (*v*/*v*), using a parafilm strip as support. The aggregates in Matrigel were incubated at 37 °C for 15 min to induce Matrigel gelification. The encapsulated aggregates were flushed into a presiliconized 15 mL spinner flask (Wheaton) using a P1000 micropipette and RPMI/B27 with insulin medium with 1% (*v*/*v*) Pen-Strep (P/S). The medium was changed every three days afterwards using RPMI/B27 with insulin and 1% P/S. Dynamic conditions were promoted using magnetic agitation set at 40 rpm.

### 4.3. Flow Cytometry

The cells were washed with PBS and the aggregates were singularized using 0.25% (*w*/*v*) trypsin-EDTA (Thermo Fisher Scientific). Trypsin-EDTA was neutralized using RPMI/20% (*v*/*v*) FBS medium. The cells were fixed using 2% (*v*/*v*) PFA for 20 min at room temperature. Then, the cells were centrifuged and resuspended in 90% (*v*/*v*) cold methanol, incubated for 15 min at 4 °C. The samples were then washed three times using a solution of 0.5% (*v*/*v*) BSA in PBS (FB1). Monoclonal mouse IgG primary antibody against cardiac troponin T (cTnT) (Thermo Fisher Scientific, Clone 13-1) or monoclonal mouse IgG primary antibody against CD31 (Dako, Clone JC70A, Nowy Sącz, Poland) were diluted in FB1 plus 0.1% (*v*/*v*) Triton (FB2) at 1:250 and 1:25, respectively, and incubated for 1 h at room temperature. The cells were then washed with FB2 and cell pellet resuspended with goat anti-mouse alexa-488 secondary antibody (Thermo Fisher Scientific) diluted 1:1000 in FB2 and incubated for 30 min in the dark. The cells were washed twice, and the cell pellets were resuspended in 500 µL of PBS and analyzed in a FACSCalibur flow cytometer (Becton Dickinson, Franklin Lakes, NJ, USA). The data were analyzed using FlowJo (v10, FlowJo LLC, Becton Dickinson, Franklin Lakes, NJ, USA).

### 4.4. Real-Time PCR

RNA from each condition and the controls was extracted using the High Pure RNA Isolation Kit (Roche, Basel, Switzerland) following the manufacturer’s instructions. RNA was quantified using a nanodrop and 1 µg of RNA was converted to cDNA using the High-Capacity cDNA Reverse Transcription Kit (Thermo Fisher Scientific). Relative gene expression was evaluated using 10 ng of cDNA, 250 nM of each primer (Table S1), and by using the Fast SYBR Green Master Mix (Thermo Fisher Scientific) with an annealing temperature set to 60 °C. Melting curves were performed at the end to assess if the primers were amplifying only the correct amplicon. The values were treated following the 2^−ΔΔCT^ method. *GAPDH* gene expression was used as an endogenous control and the relative expression was calibrated using day 0 of differentiation.

### 4.5. H&E Staining and Immunohistochemistry

The aggregates were collected and centrifuged. The pellet was resuspended in a 10% (*v*/*v*) buffered formalin solution for 48 h, dehydrated in a graded series of alcohol concentrations, immersed in xylene, embedded in paraffin, sectioned in 3 µm, and stained with Gill`s hematoxylin (Sigma-Aldrich) and eosin–floxin (Sigma-Aldrich, Burlington, MA, USA). The sections were mounted in Entellan (Merck, Rahway, NJ, USA).

Immunohistochemistry was performed using anti-CD31 (Dako, 1:30), anti-CD34 (Roche, Ready to use), anti-cTnT (Thermo Fisher Scientific, 1:50), and anti-smooth muscle actin (Dako, 1:180) antibodies. The Novolink Polymer Detection System (Leica, Wetzlar, Germany) kit was used for antibody labelling. Incubation with the primary antibodies was performed for 1 h at room temperature. Nuclei were counterstained with Mayers’ hematoxylin (Merck). Images were captured using an Olympus BX51 microscope and an Olympus DP21 Digital Camera.

### 4.6. Immunofluorescence Staining and Confocal Microscopy

The samples for confocal microscopy were prepared by transferring hiPSC-derived aggregates into Matrigel-coated 8-well µ-slides (Ibidi). After 1 h to promote light adhesion without changing the aggregates’ structure, cells were fixed with 4% (*w*/*v*) PFA for 30 min, washed with PBS, and incubated with blocking solution (10% (*v*/*v*) NGS, 0.1% (*v*/*v*) Triton-X in PBS) for 1 h. After incubation, cTnT mouse IgG antibody (Thermo Fisher Scientific, Clone 13-11, 1:250) or CD31 mouse IgG antibody (Dako, Clone JC70A, 1:25) were diluted in staining solution (5% (*v*/*v*) NGS, 0.1% (*v*/*v*) Triton-X in PBS) and incubated for 3 h at room temperature. After washing with PBS, secondary antibody goat anti-mouse IgG Alexa-488 (Thermo Fisher Scientific) was diluted 1:500 in staining solution and incubated for 2 h at room temperature. The cells were then washed once with PBS, incubated for 15 min with 3 µg/mL of DAPI diluted in PBS, washed again three times, and stored at 4 °C.

Confocal scanning laser microscopy (CSLM) and two photon excitation images were acquired using a Leica TCS SP5 confocal inverted microscope (DMI6000) with a 63.3 × water-immersion apochromatic objective (1.2 numerical aperture). Alexa-488 excitation was performed using a 488 nm line of an argon ion laser, and the fluorescence emission was collected between 500 nm and 560 nm using the tunable system and beam splitter of the Leica TCS SPC5. DAPI excitation was achieved using multiphoton excitation and a Ti:sapphire laser (Spectra-Physics Mai Tai BB, 710–990 nm) set to 780 nm as the excitation source. DAPI fluorescence emission was collected between 400 nm and 478 nm. The laser light intensity was controlled by an acoustic optical filter system. Projections (z-stacks) were obtained with 1.5 μm between slices. The images were processed using ImageJ/Fiji (http://fiji.sc) [47].

### 4.7. Calcium Imaging

The aggregates were transferred to uncoated 8-well µ-slides (Ibidi). Calcium transients were measured using the Fluo-4 Direct Calcium Assay kit (Thermo Fisher Scientific). Fluo-4 was pre-warmed at 37 °C and loaded into the cells by adding an equal volume to the culture medium present in the well. The cells were incubated at 37 °C for 30 min. In experiments using chemical receptor agonists, isoproterenol hydrochloride (Sigma-Aldrich, Burlington, MA, USA) or carbamylcholine chloride (Sigma-Aldrich, Burlington, MA, USA) were diluted in pre-warmed Fluo-4 solution and loaded into cells at a final concentration of 1 µM. The cells were incubated at 37 °C for 15 to 30 min.

The samples were measured using the confocal microscope previously described with a dry objective 10.0× *g* magnification (numerical aperture of 0.40), with images obtained at a resolution of 128 × 128 pixels, with a scan speed of 700 Hz, and with frame intervals of 97 msec during 30 to 60 s. Fluo-4 excitation was performed using a 488 nm line with an argon ion laser and the fluorescence emission was collected (500–650 nm) using a Leica HyD hybrid detector. The samples were measured within 4–5 min after leaving the incubator using a heating stage set at 37 °C to maintain the temperature before and during measurements.

### 4.8. Data and Statistical Analysis

The data analysis and statistical analysis were performed using ANOVA to assess general differences among multiple data groups and unpaired two-sided Welch’s *t*-tests to specifically compare two groups. *p*-values lower than 0.05 were regarded as statistically significant (* *p*-value < 0.05, ** *p*-value < 0.01, *** *p*-value < 0.001).

## Data Availability

The data supporting the findings of this study are available upon request.

## Supplementary Materials

The following supporting information can be downloaded at: https://www.mdpi.com/article/10.3390/ijms232415785/s1.
