# Supplementary material for: Cost-Effective Mechanical Aggregation of Cardiac Progenitors and Encapsulation in Matrigel Support Self-Organization in a Dynamic Culture Environment
Source: Int J Mol Sci. 2022 Dec 13;23(24):15785. doi: 10.3390/ijms232415785 (PMC9779514; doi:10.3390/ijms232415785)
Supplement: Supplementary file 1 [file ijms-23-15785-s001.zip › Suppl Info.pdf]

Supplementary Information

Cost-Effective Mechanical Aggregation of Cardiac Progenitors and Encapsulation in Matrigel Support Self-Organization in a Dynamic Culture Environment

Dias *et al*.

Table S1. Primer pairs used for real-time PCR.
Melting temperatures (Tm), amplicon size and primer specificity were estimated using Primer Blast.

Gene	Primers (5’ > 3’)	bp	Tm (°C)	Amplicon Size
CD31	Fwd: GCTGACCCTTCTGCTCTGTT	20	60.0	150
	Rev: TGAGAGGTGGTGCTGACATC	20	59.3	
CD34	Fwd: CCTAAGTGACATCAAGGCAGAA	22	58.0	201
	Rev: GCAAGGAGCAGGGAGCATA	19	59.5	
MYH11	Fwd: GGTCACGGTTGGGAAAGATGA	19	60.0	193
	Rev: GGGCAGGTGTTTATAGGGGTT	21	60.0	
ACTA2	Fwd: AAAAGACAGCTACGTGGGTGA	21	59.6	76
	Rev: GCCATGTTCTATCGGGTACTTC	22	58.6	
NKX2.5	Fwd: CCAAGGACCCTAGAGCCGAA	21	61.0	77
	Rev: GTCCGCCTCTGTCTTCTCCA	20	61.3	
ISL1	Fwd: GCGGAGTGTAATCAGTATTTGGA	23	60.1	102
	Rev: GCATTTGATCCCGTACAACCT	21	60.4	
TNNT2	Fwd: GTCCAAACCAAAGCCCAGGT	20	60.8	71
	Rev: CCACTCTCTCTCCATCGGGG	20	61.1	
GAPDH	Fwd: ACAACTTTGGTATCGTGGAAGG	22	60.2	101
	Rev: GCCATCACGCCACAGTTTC	19	61.7	

Video S1. Aggregates at day 15 of differentiation after mechanical formation at day 7 (Figure 1D). Scale bar: 100 µm.

Video S2. Encapsulated aggregate after culture in dynamic conditions. Scale bar: 100 µm.
